# Biological significance of miR-126 expression in atrial fibrillation and heart failure

**DOI:** 10.1590/1414-431X20154590

**Published:** 2015-08-25

**Authors:** X.J. Wei, M. Han, F.Y. Yang, G.C. Wei, Z.G. Liang, H. Yao, C.W. Ji, R.S. Xie, C.L. Gong, Y. Tian

**Affiliations:** 1 Intensive Care Unit, The People’s Hospital of Laiwu City, Laiwu City, Shandong Province, China; 2 Emergency Department, The People’s Hospital of Laiwu City, Laiwu City, Shandong Province, China

**Keywords:** MicroRNA-126, Atrial fibrillation, Heart failure, Heart function indices, N-terminal prohormone brain natriuretic peptide (NT-proBNP)

## Abstract

We investigated the biological significance of microRNA-126 (miR-126) expression in patients with atrial fibrillation (AF) and/or heart failure (HF) to examine the possible mechanism of miR-126-dependent AF and development of HF. A total of 103 patients were divided into three groups: AF group (18 men and 17 women, mean age: 65.62±12.72 years), HF group (17 men and 15 women, mean age: 63.95±19.71 years), and HF-AF group (20 men and 16 women, mean age: 66.56±14.37 years). Quantitative real-time PCR was used to measure relative miR-126 expression as calculated by the 2−ΔΔCt method. miR-126 was frequently downregulated in the 3 patient groups compared with controls. This reduction was significantly lower in permanent and persistent AF patients than in those with paroxysmal AF (P<0.05, t-test). Moreover, miR-126 expression was markedly lower in the HF-AF group compared with the AF and HF groups. The 3 patient groups had higher N-terminal prohormone brain natriuretic peptide (NT-proBNP) levels, lower left ventricular ejection fraction (LVEF), larger left atrial diameter, and higher cardiothoracic ratio compared with controls. There were significant differences in NT-proBNP levels and LVEF among the AF, HF, and HF-AF groups. Pearson correlation analysis showed that relative miR-126 expression was positively associated with LVEF, logarithm of NT-proBNP, left atrial diameter, cardiothoracic ratio, and age in HF-AF patients. Multiple linear regression analysis showed that miR-126 expression was positively correlated with LVEF, but negatively correlated with the logarithm of NT-pro BNP and the cardiothoracic ratio (all P<0.05). Serum miR-126 levels could serve as a potential candidate biomarker for evaluating the severity of AF and HF. However, to confirm these results, future studies with a larger and diverse patient population are necessary.

## Introduction

Atrial fibrillation (AF) had a prevalence of more than 2.7 million in the United States in 2010, with a 25% increase expected by 2050. However, the age-standardized prevalence of AF is 6.5 per 1000 people in China, which increases with age (1,2). In 2010, AF patients had a high rate of mortality and remaining lifetime risk, and AF was responsible for 107,335 deaths in the United states (1). With regard to heart failure (HF), 5.1 million cases are currently estimated and it is predicted to increase by 46% from 2012 to 2030 in the US. Similar to AF, HF is associated with a poor 5-year survival rate, and it accounted for 84% of overall mortality in 2010 in the United States (3,4).

AF frequently exacerbates HF, and preexisting AF is associated with a two-fold higher adjusted heart failure rate in hospitalization for HF. The combination of AF and HF is an intricate pathophysiological imbalance, which may result in worse morbidity and mortality, poorer quality of life, higher hospitalization rates, and a greater health care burden (5,6). Consequently, increasing investigations have attempted to identify a possible explanation underlying the etiology and development of AF/HF because they share many common risk factors, including advancing age, diabetes mellitus, hypertension, myocardial infarction, and valvular heart disease (7,8). In addition, abundant epidemiological data have indicated endogenous causes in the development of AF/HF, indicating that genetics may contribute to the onset of these diseases (9,10). Intensifying efforts have been made to identify potential biomarkers in early diagnosis of AF/HF. These efforts have led to the recognition of microRNA (miRNA), which participates in disease onset and progression by regulating genes known to be involved in the pathogenesis of AF/HF (11,12).

Numerous lines of evidence have suggested that miRNA plays major roles in regulating the expression of genes that are implicated in the development and pathological progression of cardiovascular diseases by controlling cell differentiation, cell death, cardiac remodeling, fibrosis, vascularization, and cardiomyocyte contraction (13–15). Studies in various models support the possible involvement of differentially expressed miRNAs in the AF/HF process, including miR-1 and miR-133, which increase arrhythmogenesis in HF by dissociating phosphatase activity (16). In addition, the endothelium-specific miR-126 (also called angiomiR-126) is located within the seventh intron of the *EGFL7* gene, which resides on human chromosome 9. This miRNA plays an important role in angiogenesis and is considered a prognostic biomarker of vascular damage and endothelial dysfunction (17). Importantly, miR-126 is also one of the most abundant miRNAs in human heart, where it may protect against the onset of atherosclerosis by inhibiting vascular cell adhesion molecule-1 protein levels during inflammation (18–20). Moreover, circulating miR-126 is downregulated in acute myocardial infarction and may have the potential for use as a novel biomarker for clinical diagnosis of acute myocardial infarction (21). Furthermore, plasma miR-126 levels are upregulated in HF patients, and miR-126 levels are negatively correlated with B-type natriuretic peptide (BNP) serum levels (22,23). Interestingly, miR-126 is also downregulated in AF patients compared with healthy controls, and may have an effect on diastolic dysfunction in AF, thus suggesting a role for miR-126 in progression of AF (11). Although a worsening of endothelial function, angiogenesis, and diastolic dysfunction can occur in HF and AF, the biological significance of miR-126 downregulation is unknown (24,25). Cellular aging or a perfusion deficiency might decrease renewal of endothelial cells or metabolic activity, which could reduce the release of miR-126 (23). This study aimed to investigate the association of serum miR-126 expression levels with development of AF/HF to determine the biological significance of miR-126 in development of AF/HF.

## Material and Methods

### Ethics statement

This study was approved by the Institutional Review Board of The People’s Hospital of Laiwu City. The study protocols complied with the Ethics Guidelines of the 1975 Declaration of Helsinki. Written informed consent was obtained from each patient.

### Study population

A total of 103 patients who were diagnosed with AF, HF, and HF plus AF (HF-AF) were admitted to the Department of Cardiology in The People’s Hospital of Laiwu City between August 2012 and February 2013. The diagnosis of AF and HF was made based on the 1994 New York Heart Association (NYHA) Functional Classification System by electrocardiogram (26). The AF group (n=35) also included nine patients with paroxysmal AF, 14 with persistent AF, and 11 with permanent AF (18 men and 17 women, mean age: 65.62±12.72 years). The HF group (n=32) consisted of 18 patients with NYHA III HF and 14 patients with NYHA IV HF (17 men and 15 women, mean age: 63.95±19.71 years). The HF-AF group (n=36) was composed of 20 men and 16 women with NYHA III-IV (mean age: 66.56±14.37 years). Our study also included 32 individuals with healthy sinus rhythm as a control group (15 men and 17 women, mean age: 58.83±13.76 years). Patients were excluded if they had a cerebral vascular accident, acute infection, chronic inflammation, severe arrhythmia, malignant tumor with chemotherapy, liver and kidney function failure, mental disorders, or pregnancy.

### Blood sample preparation

Elbow vein blood samples were acquired early in the morning after fasting. Venous blood samples (5 mL) were collected in vacuum-dried tubes with RNA-free enzymes. The samples were then separated by centrifugation at 3000 *g* for 10 min at room temperature. Serum was transferred to nuclease-free Eppendorf tubes and stored at −80°C until RNA isolation could be performed. Basic clinical data were collected from all of the participants, including medical history, history of smoking, blood pressure (BP), height, weight, body mass index, routine blood data, liver and kidney function, blood glucose levels, blood lipid levels, blood biochemical index of N-terminal prohormone brain natriuretic peptide (NT-proBNP) levels, electrocardiogram results of the lower left ventricular ejection fraction (LVEF), and X-ray examination results of the left atrial (LA) diameter and the cardiothoracic ratio.

### RNA extraction and quantitative real-time PCR

Briefly, total RNA from frozen serum samples was isolated by using a miRNA easy kit (Aidlab Biotechnologies Co., Ltd., China) and following the manufacturer's instructions. RNA levels were analyzed by using an ultraviolet spectrophotometer (Thermo Co., Ltd., USA) to measure the absorbance at 260 and 280 nm. RNA samples exhibited high purity at A_260/280_>1.9. The resulting miRNA was retained for quantitative real-time PCR (qRT-PCR). For qRT-PCR, 5 μg of total RNA was reverse-transcribed to synthesize cDNA using the GoScript™ Reverse Transcription System (Promega Co., Ltd., USA) according to the manufacturer’s instructions. Briefly, the reactions involved incubations at 25°C for 5 min, 42°C for 60 min, and 70°C for 15 min, and were then maintained at −20°C until further study. The qRT-PCR was performed using GoTaq qPCR Master Mix (Promega Co., Ltd.) with the following conditions: incubation for 2 min at 95°C, followed by 40 cycles of annealing at 95°C for 15 s, and extension at 60°C for 60 s. The miR-126 PCR primer was designed by Ribobio Co., Ltd. (China). Primers used in this study were as follows: miR-126, 5′-TCGTACCG TGAGTAATA ATGCG-3′; and U6 snRNA, 5′-CTCGCTTCGGCAGCACA-3′.

### Data analysis

Expression of miR-126 was measured with the 2^−ΔΔCt^method and the level of human U6 snRNA was used as an endogenous reference (27). Relative expression of miRNA-126 is reported as miRNA-126 relative to an internal control gene, called ΔCt. The ΔCT and ΔΔCT values were calculated using the following mathematical formulas: ΔCt=Ct_miRNA-126_ − Ct_U6_ and ΔΔCt=ΔCt_case_ − ΔCt_control_.

### Statistical analysis

Data are reported as means ± SD. Statistical analysis was performed using SPSS 18.0 software (SPSS Inc, USA). Comparisons between two groups were performed with the independent-sample *t*-test. Multiple group comparisons were assessed with analysis of variance followed by Bonferroni’s or Fisher’s LSD *post hoc* tests. Pearson’s correlation analyses between different indices were carried out. P<0.05 was considered to be statistically significant. We also used multiple linear regression analyses to evaluate the independent associations of miR-126 with cardiac function index parameters after adjusting for differences in age, diabetes, drug use, and other confounding factors.

## Results

### General clinical data and biochemical indicators

Differences in the general clinical data among the AF, HF, HF-AF, and control groups are shown in Table 1. There were no significant differences in age, gender, BP, a history of hypertension, or a history of diabetes mellitus among the four groups (all P>0.05). Moreover, there was no significant difference among the groups when treated with different basic drugs (all P>0.05). We also examined differences in biochemical indicators among the four groups. There were no significant differences in levels of aspartate aminotransferase, alanine transaminase, creatinine, glucose, white blood cells, total cholesterol, triglycerides, low-density lipoprotein cholesterol, and high-density lipoprotein-cholesterol among the four groups (all P>0.05, Table 2).

**Table:**
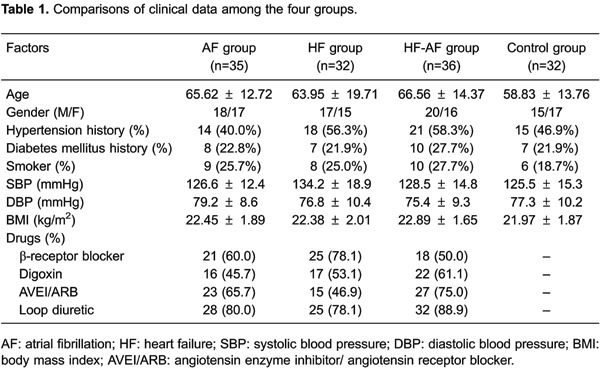

**Table:**
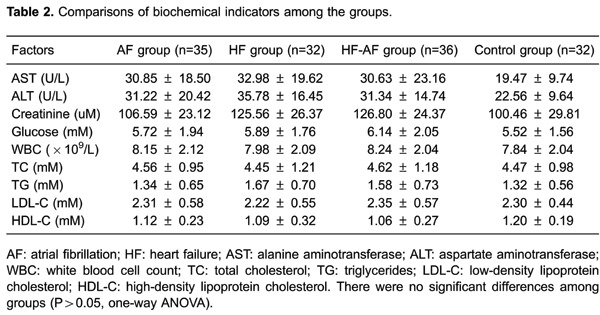

### Expression of miR-126

Relative expression of miR-126 was lower in the AF group than in controls (P<0.01). Moreover, we found that miR-126 expression was significantly lower in patients with permanent or persistent AF than in those with paroxysmal AF (all P<0.05). We also found that miR-126 was frequently downregulated in the HF group compared with the control group (P<0.01). Further analysis was performed to compare relative miR-126 levels between NYHA class III and IV HF patients. Slightly lower miR-126 levels were observed in NYHA class IV HF patients than in class III HF patients, but this difference was not significant (P>0.05). Lower miR-126 levels were observed in the HF-AF group than in controls (P<0.01). Additionally, miR-126 expression was markedly lower in the HF-AF group (than in the AF group (P<0.01) and the HF group (P<0.01, Table 3).

**Table:**
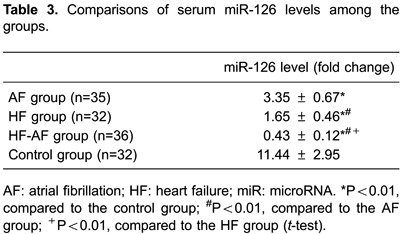

### Comparison of cardiac function indices

NT-proBNP, LVEF, LA diameter, and the cardiothoracic ratio were assessed among patients in the AF, HF, HF-AF, and control groups. NT-proBNP levels were significantly higher in the AF group (P<0.01), HF group (P<0.01), and HF-AF group (P<0.01) compared with controls. Patients in the AF group had lower NT-proBNP levels than those in the HF group (P<0.05). In addition, NT-proBNP levels in the AF group were significantly lower than those in the HF-AF group (P<0.05).

We found that LVEF was higher in controls than in the AF (P<0.01), HF (P<0.01), and HF-AF groups (P<0.01). Additionally, LVEF was higher in the AF group than in the HF and HF-AF groups (both P<0.05). The LA diameter was larger in the AF (P<0.01), HF (P<0.01), and HF-AF groups (P<0.01) than in the control group. A similar difference was found in the cardiothoracic ratio between the AF, HF, HF-AF, and control groups (all P<0.01). However, the cardiothoracic ratio showed no significant difference among the AF, HF, and HF-AF groups (all P>0.05, Table 4).

**Table:**
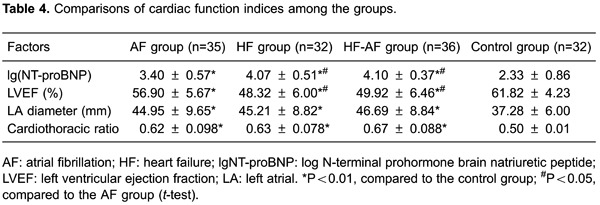

### Correlation analysis

Correlations among NT-proBNP levels, LVEF, LA diameter, and the cardiothoracic ratio with miR-126 expression were performed using Pearson correlation analysis. We found that miR-126 expression was negatively associated with LVEF (r=0.374, P<0.01) and the logarithm of NT-proBNP (r=−0.783, P<0.01). Similar correlations were also observed in the LA diameter (r=−0.517, P<0.01) and the cardiothoracic ratio (r=−0.587, P<0.01). We also found that serum miR-126 levels were negatively correlated with age in HF-AF patients (r=−0.31, P<0.01, Table 5).

**Table:**
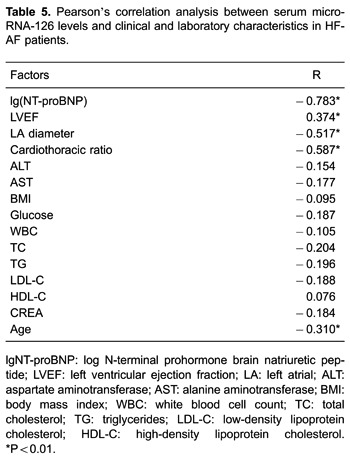

### Multiple linear regression analyses

After adjustment for confounding factors (gender, age, diabetes mellitus, and smoking), serum miR-126 expression levels were positively correlated with LVEF, and negatively correlated with the logarithm of NT-proBNP and the cardiothoracic ratio (all P<0.05, Table 6).

**Table:**
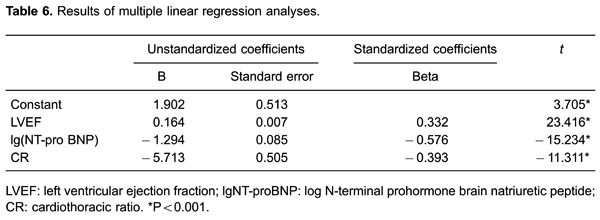

## Discussion

The prevalence of AF is positively correlated with the severity of HF. AF may induce or aggravate HF, and vice versa (28). In recent years, evidence has shown that the mortality of HF-AF patients remains high, and that AF, HF, and HF-AF patients have a poor prognosis (5). Accumulating evidence has suggested that miRNAs are closely related to the progression and prognosis of AF and HF, but the underlying mechanisms are still not completely understood (29,30). Therefore, we aimed to examine serum miR-126 expression levels in patients with AF and/or HF, and to investigate the potential of circulating miR-126 as a serum biomarker in patients with AF and/or HF. We found that serum miR-126 levels in patients in the AF, HF, and HF-AF groups were lower than those in the controls. Notably, miR-126 serum levels were lowest in HF-AF patients among the four groups. These results indicate that circulating serum miR-126 levels are related to the development, progression, and severity of AF and HF.

MicroRNA-126 is a short non-coding RNA that is derived from the *EGFL7* gene (31). Numerous studies have shown that miR-126 levels are highly expressed in vascularized tissues, including the heart, liver, and lungs, and is specifically expressed in endothelial and hematopoietic cells (32,33). Expression levels of miR-126 are mediated by Kruppel-like factor 2a (*klf2a)*, a mechanosensitive zinc finger transcription factor, and may lead to activation of the vascular endothelial growth factor signaling pathway in the endothelium (34). Additionally, miR-126 knockdown may result in impaired endothelial cell migration during the processes of vessel growth, development, and organization, which are closely related to the development of AF and HF (35). Therefore, miR-126 could play an important role in the regulation of vascular endothelial growth factor pathway activation. Consequently, abnormal miR-126 expression levels in serum may induce defective angiogenesis and result in an increased risk of AF and HF. Furthermore, decreased miR-126 expression is associated with the outcome of patients with chronic HF, and it could be helpful in the diagnosis of chronic HF (36).

Another important finding in our study was that patients in the AF, HF, and HF-AF groups had higher NT-proBNP levels, a lower LVEF, a larger LA diameter, and a higher cardiothoracic ratio than the controls. These results suggested that heart function indices, including NT-proBNP levels, LVEF, LA diameter, and the cardiothoracic ratio, were closely associated with development and progression of AF and HF, which were significantly correlated with the severity of HF and AF. A previous study reported that NT-proBNP expression could be used as a risk parameter in AF/HF because its expression was positively correlated with an increased risk of HF, stroke, and mortality (37). In addition, NT-proBNP levels are significantly higher in patients with AF than in controls, and thus could serve as a helpful serum biomarker to effectively evaluate heart function in heart diseases (38). To better understand the correlations between serum miR-126 levels and heart function indices, we carried out Pearson’s correlation analysis to explore the underlying mechanism. This analysis showed that miR-126 serum levels were negatively correlated with LVEF, while they were positively associated with the logarithm of NT-proBNP, LA diameter, and the cardiothoracic ratio. Previous studies have shown that miR-126 regulates the pathological processes of myocardial hypertrophy, myocardial fibrosis, and changes in myocardial ion channels (33,39), which are significantly associated with heart diseases, including AF and HF. In this respect, decreased serum miR-126 levels may underlie changes in heart function indices, and then induce development of AF and HF.

In conclusion, our study shows that serum miR-126 expression levels in patients with AF, HF, and HF-AF are low, especially in those with HF-AF. Moreover, serum miR-126 levels are closely correlated with heart function indices, including NT-proBNP levels, LVEF, LA diameter, and the cardiothoracic ratio. However, the potential limitations of the use of U6 snRNA as an endogenous reference may have affected detection of plasma miRNA expression. Collectively, our data indicated that miR-126 serum levels could serve as a potential biomarker for evaluating the severity of AF and HF. Future studies with a larger number of patients from other populations are necessary to confirm these results.
